# Quality Control of Next-Generation Sequencing-Based HIV-1 Drug Resistance Data in Clinical Laboratory Information Systems Framework

**DOI:** 10.3390/v12060645

**Published:** 2020-06-14

**Authors:** Rupert Capina, Katherine Li, Levon Kearney, Anne-Mieke Vandamme, P. Richard Harrigan, Kristel Van Laethem

**Affiliations:** 1 National HIV and Retrovirology Laboratories at JC Wilt Infectious Diseases Research Centre, Public Health Agency of Canada, Winnipeg, MB R3E 3L5, Canada; rupert.capina@canada.ca (R.C.); lik3456@myumanitoba.ca (K.L.); 2 Scientific Informatics Services at National Microbiology Laboratory, Public Health Agency of Canada, Winnipeg, MB R3E 3R2, Canada; lev.kearney@canada.ca; 3 Rega Institute for Medical Research, Department of Microbiology, Immunology and Transplantation, KU Leuven, 3000 Leuven, Belgium; annemie.vandamme@kuleuven.be; 4 Center for Global Health and Tropical Medicine, Unidade de Microbiologia, Instituto de Higiene e Medicina Tropical, Universida de Nova de Lisboa, 1349-008 Lisbon, Portugal; 5 Department of Medicine, University of British Columbia, Vancouver, BC V6T 1Z3, Canada; richard.harrigan@ubc.ca; 6 AIDS Reference Laboratory, University Hospital Leuven, 3000 Leuven, Belgium

**Keywords:** laboratory information systems, HIV-1 drug resistance, next-generation sequencing, quality control, quality management

## Abstract

Next-generation sequencing (NGS) in HIV drug resistance (HIVDR) testing has the potential to improve both clinical and public health settings, however it challenges the normal operations of quality management systems to be more flexible due to its complexity, massive data generation, and rapidly evolving protocols. While guidelines for quality management in NGS data have previously been outlined, little guidance has been implemented for NGS-based HIVDR testing. This document summarizes quality control procedures for NGS-based HIVDR testing laboratories using a laboratory information systems (LIS) framework. Here, we focus in particular on the quality control measures applied on the final sequencing product aligned with the recommendations from the World Health Organization HIV Drug Resistance Laboratory Network.

## 1. Introduction

HIV drug resistance (HIVDR) has become a major challenge as the use of antiretroviral therapy (ART) continues to increase worldwide, due to the high mutation rate of HIV, the incredible intra-host diversity of HIV, and selection pressure for drug-resistant HIV virions during ART [1,2]. In 2017, the World Health Organization reported that 5–28% of individuals on ART and 50–90% of individuals failing ART showed non-nucleoside reverse-transcriptase inhibitor (NNRTI) resistance, additionally calling the fight against antimicrobial resistance a global priority [3]. The standard methodology for HIVDR genotyping has been Sanger sequencing, which generates a single consensus sequence using a detection threshold of around 15–20%; however, this prevents detection of minority resistant variants below this frequency threshold [2,4]. The presence of minority resistance variants holds clinical significance as it can both increase the potential for virological failure and hinder immune system recovery [4]. Additionally, minority variants can lead to the accumulation of drug resistance mutations, further increasing the risk of exhausting treatment options [4]. In contrast, next-generation sequencing (NGS) technologies have increased sensitivity and resolution for the detection of HIV quasispecies and minority variants in a more time- and cost-efficient, and scalable manner [2,4].

With the advantages of NGS comes the need for comprehensive quality standards, as NGS for clinical applications can be affected by error or bias at a variety of stages [5]. There are numerous steps of HIVDR assays that can implement quality control measures, such as nucleic acid extraction, cDNA synthesis, PCR, library preparation and sequencing, assembly, and variant calling. Many clinical labs use software or bioinformatics pipelines to perform sequence analysis and as such, validation of the pipeline in use is necessary to ensure the test can reliably detect variation [6,7]. As HIVDR testing continues to become common practice for guiding ART regimes, clinical labs need to maintain both internal and external quality control measures, as well as a uniform standard of quality assurance [5,8]. The use of modern technologies such as NGS continues to drive massive data production, creating a need to systematically organize both clinical and quality control results, while flagging potential problems that could impact data quality.

Quality management in a clinical lab encompasses several components, including quality control (QC), quality assurance (QA) and external quality assessment (EQA). QC refers to procedures that monitor and evaluate each step of a workflow, ensuring that the resulting sequences are accurate and flagging those that break pre-defined rules [7,9]. Levey–Jennings control plots are commonly used in clinical labs to set control limits for monitoring variability in QC data [10]. These plots are often applied with Westgard or Nelson rules, which implement either individual or multi-rule procedures to define the criteria for violation during data analysis, effectively minimizing false rejections while maximizing true error detection [10,11]. In terms of HIVDR testing, appropriate QC measures can ensure that all sequence data used to generate patient reports are accurate and meet the required laboratory standards for flagging risk of ART failure [7]. QA refers to an established, continuous process employing both corrective and preventative measures to provide confidence that quality standards will be met [9]. Further QA procedures are often used to reduce risk of errors or contamination in clinical testing, such as confirmatory tests with previously established gold standard methods [7]. EQA is the use of proficiency tests often sponsored by a formal provider that assesses lab performance using pre-established criteria, allowing for interlaboratory comparison of results [9]. While both QA and EQA programs are important in clinical settings, here we focus on implementing QC strategies into the HIVDR testing workflow and how these programs can be organized and maintained using a Laboratory Information System (LIS).

Over the last decade, regulatory bodies have established quality control guidelines specific to NGS-based protocols. The Clinical and Laboratory Standards Institute (CLSI) and the U.S. Food and Drug Administration (FDA) have established guidelines for quality management systems that are widely used in public health laboratories performing clinical diagnostics [7,12]. Both the 2014 update of the CLSI MM09-A2 document and the 2016 FDA guidance draft highlight regulations for NGS methods in clinical testing as compared to traditional Sanger-based assays [9,12]. These documents specifically address important QC steps to identify sequencing artefacts, low quality base calls, and poor alignments, as well as device and performance validation. Similarly, in the Winnipeg Consensus, Ji et al. emphasize the need for standardization of NGS HIVDR pipelines to produce consistently high-quality sequence data, and highlight the five key components of a reliable NGS HIVDR pipeline as NGS read quality control and assurance, NGS read alignment and reference mapping, HIV variant calling and variant quality control, HIVDR interpretation and reporting, and analysis data management [2]. More recently, in November 2019, the FDA approved the first NGS assay for the detection of HIV-1 drug-resistant mutations for marketing in the United States [13]. The Sentosa SQ HIV Genotyping Assay by Vela Diagnostics USA Inc. reports a sensitivity and specificity higher than 95% for detecting 342 HIV drug resistance mutations and is intended for HIV-1 infected patients who are preparing for or already taking ART.

While there is extensive coverage of quality control procedures for sample processing in NGS and specifically HIV-sequencing, the data produced by such techniques provide multiple opportunities for continued quality control of the final sequence [2,7,8,14]. The integration of an LIS allows for the management of samples and their associated data while automating workflows and incorporating instrument specifications, ultimately allowing the user to view all data associated with an assay as a package within a centralized program [15]. While a laboratory information management system (LIMS) program generally refers to application within large-scale public health systems such as national reference laboratories, an LIS indicates a more definitive tier such as clinical laboratories handling patient-specific specimens [16]. By employing systems such as an LIMS or LIS to monitor batch and monthly or quarterly QC trends, clinical laboratories can maintain a high standard of quality management. Here, we present a comprehensive outline of quality control strategies for clinical NGS-based HIVDR testing. This includes numerous QC steps that can be incorporated during both sample- and data-processing. Finally, we detail how an extensive QC program can be orchestrated within the framework of a laboratory information system, which we believe is critical to long-term success.

## 2. Quality Control Management with Laboratory Information Systems

High-throughput technologies, such as NGS, are challenging for clinical laboratories because of their massive data production and the complexity of result interpretation. Data management and bioinformatics strategies need to be incorporated in an LIS for the data to become consistently useful in a clinical setting. Specimen identifiers, relevant metadata, and results need to be able to transition between instruments, bioinformatics pipelines, and an LIS seamlessly without reproducing data. In addition, these systems must work together to provide efficiencies to the testing workflow, such as evaluating controls, reflux testing, real-time updates of test status, associate equipment and reagent information for future analysis and quality control efficiencies. The core function of an LIS can be split into three categories according to the Association of Public Health Laboratories: pre-analytical, analytical, and post-analytical [16].

### 2.1. Pre-Analytical

Specimen, patient and test management and tracking are critical in the pre-analytical phase. Specimen tracking systems and, where feasible, barcoding, reduces the chance of sample swapping. Flags for priority, correct specimen types for testing and appropriate metadata needed for testing can be incorporated at this stage and follow the sample through processing. Electronic health systems (ehealth) have greatly reduced the number of data entry errors by allowing clients to electronically submit orders for testing via standardized protocols. Patient management utilizes an LIS to audit or restrict who views, enters, or edits patient information, which alleviates privacy concerns and ensures the safekeeping of personal records. These records can then be anonymized and associated with sample submissions. Health care information security systems need to preserve the confidentiality of health records, especially when people living with HIV can be convicted for criminal offenses in some countries for not disclosing their HIV status to their sexual partners [17].

### 2.2. Analytical

The analytic phase focuses on interfacing with specimen handling and instrument software (middleware) for streamlined processing [18]. This includes coordinating batch testing across instruments and assigning reagent lot numbers to particular batches. The LIS can also be used to record test results and quality control metrics, while flagging specimens that may need repeat testing.

#### 2.2.1. Reagent Tracking and Inventory

Different reagent lot numbers may perform differently, sometimes compromising test results. Therefore, it is crucial that new lot numbers are validated before use for clinical testing. Lot numbers and expiration dates are often recorded in paper form or in a stand-alone database that makes tracking and monitoring the performance of reagents difficult. In the event of a vendor recall, identifying affected batches and associated samples may prove to be challenging. An LIS can store information on reagent lot numbers and associated batches/samples to simplify this type of investigation. Some vendors have lot numbers encoded in barcodes to allow the seamless import of reagent information into an LIS.

#### 2.2.2. Instrument Integration and Automation

To reduce data entry errors, data should ideally not be transcribed from stand-alone instruments. Instead, it should be input by interfacing these instruments with a secured network and using messaging protocols to move the data automatically into centralized repositories. A centralized LIS for data storage and data analysis has a number of advantages. In addition to keeping relevant metadata and patient information linked to results, it reduces the amount of redundant data stored in multiple locations. This not only decreases the amount of infrastructure needed to store and organize these data, but increases security by limiting the number of points of entry where a possible data breach could occur. Additionally, database interoperability (capability of different software to exchange data) allows data to transcend between systems without human intervention, which allows independent tools to work together as a system. With this strategy, systems can operate constantly and relatively independently of technician hours. Automated data transfer also prevents the need for technicians to waste time moving information such as specimen data, raw data, or test results between systems. By utilizing Representational State Transfer Application Program Interface (REST API), scripting to automate the execution of tasks, database views (searchable objects in a database), and queries to view the data as required eliminates downtime and wasted staff hours and also improves data quality. Having a centralized tool ensures that every piece of equipment has access to the most current information that it needs for its function. In the event that amendments need to be made to sample or test data, the update can be applied to the database and propagated across all systems. This ensures that all systems are using the most accurate information and eliminates the possibility of conflicting information being present in different functioning databases.

#### 2.2.3. Quality Control Checks and Tractability

**Figure 1:**
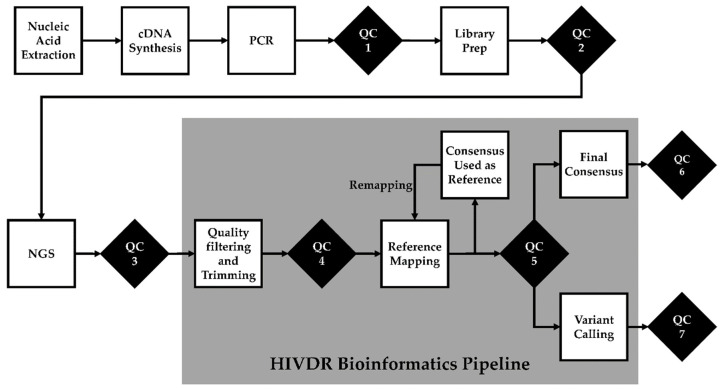
Quality control (QC) checks in NGS-based HIV drug resistance testing. QC1: post-PCR quality check. QC2: library preparation quality check. QC3: post-sequencing run quality check. QC4: bioinformatics pre-processing quality check. QC5: post-reference mapping quality check is performed only after the final remapping. QC6: cross-contamination quality check. QC7: “bad” mutation quality check.

The ability to automate quality control checks is another key component of a LIS. Automated warnings can be provided to technicians to indicate equipment is operating outside of control limits, or that data output is outside of specification limits. These warnings can trigger an investigation by the operator. Further, programmed pass/fail criteria can allow data to move to the next step of the assay without human intervention (Figure 1). This is useful for computationally demanding analyses where you want to review sequencing controls prior to processing data in a high-performance computing cluster. In the event of failed testing, repeat testing can be ordered with increased priority to meet the required turnaround time. Metrics and thresholds at each QC checkpoint summarized in Table 1 are general guidelines, as different NGS platforms and protocols may have their own quality control parameters.

##### QC Checkpoint 1: Post-PCR Amplification

To increase turnaround times in HIVDR testing, post-nucleic acid extraction and –cDNA synthesis quality control checks are usually not directly assessed. It is nearly impossible to visualize or quantify HIV at this point. Batched tests are first assessed after PCR is performed using agarose gel or capillary electrophoresis or other methods. As in any scientific assay, appropriate negative and positive controls must be included and monitored throughout the process and carried through to sequencing. External controls can be purchased through third party vendors, such as ACCURUN (SeraCare, Milford, MA, USA) and AcroMetrix (Thermo Fisher Scientific, Grand Island, NY, USA) molecular controls, where specific reagent lots can be reserved to minimize lot-to-lot variations. If amplification is observed in negative controls, the entire batch is deemed a failed test and corrective action needs to be taken, such as evaluating contamination of reagents and specimens. If the amplicon is not observed for positive controls, this would typically be considered a failed test. Most samples with adequate input HIV copy numbers would be expected to successfully be amplified. The importance of having an LIS is that rather than considering each “successful” run or sequence as an independent event, tracking these data longitudinally allows the lab to monitor trends over time, and link these trends with other data and with specific lots, staff, changes in methods or other factors. For example, samples with low viral loads begin to have an increasing failure rate; this can be a signal that something is amiss far before a positive control with a higher viral load begins to show problems.

##### QC Checkpoint 2: Library Preparation

After mechanically or enzymatic fragmentation, and subsequent library preparation, the distribution of fragment size can be evaluated using capillary electrophoresis and/or DNA quantitation. Electropherograms should reveal a single narrow peak (for example, between 300 and 500 bp) with limited tailing, broadening, or primer dimers. While this quality check is important during method development and validation, it is also critical to monitor the performance of the many steps of library preparation. Again, storing these results in an LIS allows the monitoring of trends in success or failure. For example, without tracking the progress of a sample through the testing system, it might be extremely difficult to work out that one half of a thermocycler has failed. However, if the sample is monitored for its journey through different instruments (and ideally, the locations within these instruments), these issues can be readily identified and addressed.

##### QC Checkpoint 3: Post-Sequencing Run

Quality control checks after a sequencing run are vital for monitoring the health of the sequencer and performance of the sequencing run. For Illumina sequencing, the Sequence Analysis Viewer (SAV) (Illumina, San Diego, CA, USA) can be used to evaluate post-run metrics such as total yield, cluster density, proportion of clusters passing filter, percentage of PhiX control, and base quality scores. For expected values, see Hutchins et al. 2019 [8].

##### QC Checkpoint 4: Pre-Processing

High-quality sequencing data are necessary for accurate downstream analyses. High throughput sequencing often uses barcodes (or unique indexes) to multiplex samples in a single sequencing run to reduce cost and time. Following a run, the pooled data must be demultiplexed by reading the barcode of each read and binning it with other reads derived from the same sample.

The quickest way to monitor the quality of demultiplexed sequencing data is to assess the total number of reads per sample [19], which can be accomplished with the MiSeq Reporter software (Illumina, San Diego, CA, USA). Usually, a minimum number of reads is needed to continue analyses, which for HIVDR falls around 10,000 reads. When investigating issues with demultiplexing, the list of barcodes found by the Miseq Reporter can be compared to the expected barcodes. Poor demultiplexing may be a result of barcode sequences being entered incorrectly into the sample sheet or poor barcode read quality [20].

Following demultiplexing, HIVDR pipelines take advantage of several pre-preprocessing tools to clean data. Early data-processing includes adapter clipping, quality filtering and trimming, merging paired-end reads, removing duplicates, and normalization [21,22,23,24,25,26,27,28]. It has been recommended at the very least that low-quality reads (Q < 25) and short reads (<75 bp) should be excluded from downstream analyses for HIVDR [2]. It is important to track in an LIS how many reads are dropped during pre-processing, which may indicate a potential issue with the run. After pre-processing, the quality of sequence reads can be assessed with software such as FastQC (Babraham Institute, Cambridge, UK) before analysis with a HIVDR pipeline [29]. Certain quality metrics should be recorded, such as per-base quality score, average read quality scores, sequence length distribution, per base N content, sequence duplication levels, overrepresented sequences, adapter content, and kmer content, which all have been thoroughly reviewed [8]. While FastQC gives three levels of quality indicators for each metric (good, warning, and fail), a failed metric may not necessarily mean a failed run given the context of the experiment.

##### QC Checkpoint 5: Post-Reference Mapping

At this quality control checkpoint, it is highly recommended that reference mapping metrics are evaluated after the final remapping is performed. Normally, the percentage of genome coverage, in combination with the percentage of reads that align to the reference and depth, will give an indication of mapping performance [8]. In the case of HIVDR genotyping, 54.2% the pol gene needs to be covered. More specifically, codons 10 to 93 in PR, codons 41 to 238 in RT, and codons 51 to 263 in IN are the minimal coverage usually needed for approved HIVDR testing [30]. Certain pipelines, such as HyDRA (Public Health Agency of Canada, Winnipeg, MB, Canada), have built in a QC metrics for variant calling. HyDRA requires a minimum of ≥ 5 allele counts, a Q score of at least ≥ 30, and a depth of ≥100 at each loci to perform variant calling [2,31]. Read depth can be increased by iterative mapping, whereby the consensus sequence generated by the first round of alignment is used as a reference genome for a subsequent round of reference mapping. Another metric for reference mapping is the evenness of coverage—the uniformity of read distribution across a reference genome. Certain library preparation methods are more likely to create a bias, which affects the accuracy of variant calls leading to false negatives. If it is not built in the HIVDR pipeline, visual uniformity checks can be performed software like Geneious (Biomatters, San Diego, CA, USA), Tablet (The James Hutton Institute, Aberdeen, UK), or UGENE (Unipro, Novosibirsk, Russia). Non-uniformity can also be calculated as the variance of sequencing depth.

##### QC Checkpoint 6: Sample Mislabeling and Contamination

Molecular diagnostics that rely on PCR to amplify low-copy DNA fragments from clinical specimens are extremely sensitive to contamination [32]. Sources of contamination can originate from specimens processed from the same or previous batches, lab strains, or even lab personnel. Quality control procedures during pre-testing (e.g., laboratory set-up and SOPs), specimen processing (e.g., unidirectional workflow and inclusion of internal controls), and post-testing (e.g., sequence evaluation) are important to limit cross-contamination.

A form of cross-contamination is the physical carry-over of material from one sample into another during RNA extraction, cDNA synthesis, PCR amplification, or library preparation. If the two samples are distally related, a consensus sequence derived from this type of contamination can harbor an unusually high number of nucleotide mixtures (i.e., R, W, S, and G for two-nucleotide mixtures; B, D, H, V, and N for more than two). If >3.5% of nucleotide positions in the consensus sequence (20% cut-off) are mixtures, the sample should be flagged for further investigation and re-testing [33]. Additionally, to identify contamination, all sequences in a batch can be cross-checked for genetic relatedness, and be further compared to other recent batches and lab strains. Pairwise comparisons of genetic distances between each consensus sequence generated from the samples can be conducted using the WHO HIVDR QC Tool (University of British Columbia, Vancouver, BC, Canada) [34]. Sequences derived from lab strains used for research, assay validation, or as positive controls should be included in regular QC checks. Positive controls should also be sequenced and analyzed in this step. If a positive control contains contaminating sequences and displays divergence from previous runs, the entire run will need to be discarded and reprocessed.

In a situation where a sample tube has been mislabeled or if two or more samples have been switched during processing, the sequence may appear to be “normal”, without a high proportion of ambiguous bases. The only way to identify sample mix-ups is by comparing the sequence from the sample in question to the historic sequences derived from the same patient. Clinical laboratories have the ability to compare sequences from the same patient taken from different timepoints throughout their treatment cascade. Pairs of sequences derived from the same patient with ≥2.5% genetic dissimilarity may be a result from a sample mix-up or mislabeling and should be flagged for further investigation and re-testing. Pairs of sequences from different patients or from lab strains with <0.5% genetic dissimilarity should also be flagged for re-testing [35,36]. In this case, epidemiological linkage needs to be taken into consideration by checking with clinicians or public health officers. It is therefore important for a LIS to keep a repository of previously obtained sequences as well as lab strain sequences to check for contamination.

While genetic pairwise comparisons of a large number of consensus sequences is a quick approach to detecting anomalies, it is not sensitive to detecting traces of contaminating reads that contribute to variant calling. In NGS, filtering for contamination is critical in sequencing analysis. Low level spillover from one sample to another, or traces of “index-hopping”, can both occur in high throughput sequencing [37]. While removing small numbers of contaminating reads is ideal before moving forward with variant calling [38,39], this may hide underlying causes for the cross-contamination, such as faulty instruments or under-performing operators. Contaminating reads can be detected using ViCroSeq (IrsiCaixa, Badalona, Spain), a tool that can check cross-contamination between different samples from the same batched run [40]. Initially, this tool uses paired-end or single-end FASTQ reads to align to HXB2 as a reference. In a pairwise fashion, NGS reads from one sample are then mapped to the consensus sequence of other samples within the same batch of sequencing run. Contaminating reads are detected when they have much better mapping metrics to other sample’s consequences sequence.

A particularly helpful measure to monitor longitudinally using the LIS is the proportion of apparently “mixed” bases present in a known clonal positive and/or repeated clinical sample. If it appears that the number of low-level mixtures reported in such a control is creeping upwards or downwards over time (or varies greatly in a single batch), the data may be suspect.

##### QC Checkpoint 7: “Bad” Mutations

Evaluation of post-run sequences is an important step to ensuring good quality data for a drug-resistance report. In this step of quality control, excessive “unusual” amino acid mutations and APOBEC hypermutations are detected using Stanford University’s HIVdb-NGS (Stanford University, Palo Alto, CA, USA), in which local LIS can connect to via Sierra Web Service [41]. HIVdb-NGS accepts codon frequency tables from protease, reverse transcriptase, and/or integrase in the file format CodFreq or AAVF [2,41]. Unlike the WHO BCCFE HIVDR QC Tool, this program analyzes the number of unusual mutations and hypermutations at eight different mutation detection thresholds (0.1%, 0.2%, 0.5%, 1%, 2%, 5%, 10%, and 20%). The default is set as a Sanger-like detection threshold at 20%. High numbers of unusual mutations and hypermutations detected at lower thresholds may be artefacts from PCR errors and run the risk of cumulatively contributing to variant calls. They are difficult to interpret because they cannot be confidently identified without the use of unique molecular identifiers or a primerID for each virus template. The results returned from lower detection thresholds can help users identify and investigate artefactual mutations. Unusual mutations are amino acids with low prevalence (<0.01%) in published Sanger HIV-1 Group M sequence database that are not signature APOBEC hypermutations [42]. When HIVdb-NGS identifies unusual mutations >1% of the total reads at a given threshold, the program suppresses drug resistance interpretation. The presence of these types of mutations may be an artefact resulting from equipment failure or PCR error, and requires retesting.

APOBEC-mediated hypermutations are disproportionately high frequency of guanine to adenine transitions in HIV provirus caused by host gene-editing enzymes, APOBEC3G and APOBEC3F [43]. They can be easily detected in samples such as whole blood or dried blood spots and plasma samples contaminated with proviral DNA [44]. Hypermutations highly skews HIV’s rate of evolution, when in fact, their apparent sequence divergence is an artefact. Along the genome, they change tryptophans to premature stop codons, which causes the production of a replication-incompetent virus [43,45,46,47]. They also generate apparent mutations in pol that are associated with drug resistance in a live virus. Because of this, it is critical that the presence of APOBEC hypermutations and premature stop codons are flagged for further investigation. When HIVdb-NGS identifies more than one DRM-associated hypermutation, it automatically suppresses drug resistance interpretation due to failed quality control assessment.

Detecting insertions or deletions (indels) is a critical QC step, as a frameshift caused by an indel result in a replication-incompetent virus. Codon indels may not be considered a fail, but they need to be confirmed as they do not occur frequently in pol. One insertion in RT, T69i, confers a high level of resistance towards all NRTIs [48]. NGS-based HIVDR genotyping pipelines that rely only on reference mappers to generate variant calls are still inadequate regarding indel detection [2]. One strategy is to use contigs from de novo assembly as a reference sequence for read mapping [49,50]. These pipelines have yet to be validated for HIVDR genotyping, but may be adequate as a QC tool for detecting insertions and deletions.

##### Clonal and Repeated Sample Check

**Figure 2:**
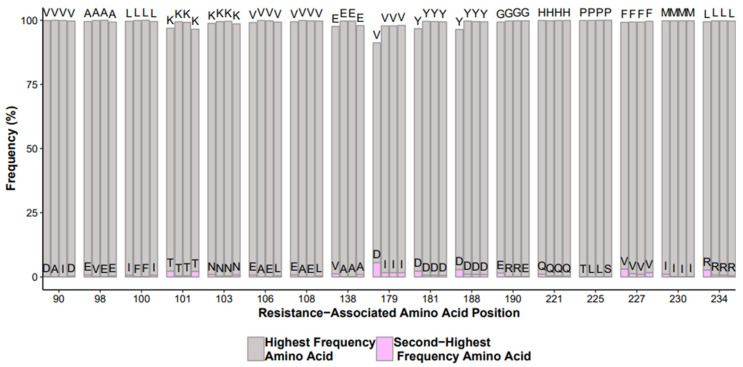
Example output of NNRTI mutations from control runs over time with highest frequency amino acid (gray) and variant (pink).

Infectious molecular and repeated samples give insights on how well the overall HIVDR test is performing. HIV-1 infectious molecular clones are a laboratory-generated and fully characterized full length genome sequence, albeit at the bulk population level. Samples that have gone through testing can be retested and can serve as a positive control. One can monitor each step of the HIVDR workflow by assessing these types of “positive controls” and evaluate the batch effects, or how each batch deviates from the expected values (Figure 2). Certain strategies should be implemented such as diluting a lab strain into different viral loads to monitor how well the overall HIVDR tests are performing with varying input copy numbers. These positive controls obviously need to be positive in a PCR test. If a low-copy-number positive control happens to be PCR-negative, one cannot expect to efficiently amplify clinical samples with low viral loads or drug resistant variants at lower frequencies in clinical samples. It is also recommended to monitor the accuracy of the variant calls among the sequences derived from the lab strain with varying viral loads. While infectious molecular clones are “clonal”, it is expected for them to harbor low abundant mutations generated from viral expansion during tissue culture.

While evaluating clonal and re-tested samples, it is also good practice to check all mutations that occur within or adjacent to repeating bases. Certain drug resistant mutations, such K65R and K103N in RT, occur in poly-A sites and could be problematic for cDNA synthesis and PCR regarding enzyme slippage, accuracy of the basecall, and/or difficulty with read alignment. Different ratios of plasmids containing these mutations (e.g., 50:50, 80:20, 90:10, 95:5, and 99:1) can be introduced into the PCR step to evaluate the batch’s performance in accurately calling the mutation, however this ignores the nucleic acid extraction and cDNA synthesis steps [31]. As mentioned above, sequences derived from positive controls should be included in every batch of sample mislabeling and cross-contamination check. Different sequence results indicate a sample mix up. The presence of excessive unusual mutations and APOBEC hypermutations in infectious molecular clones and plasmids indicate the issue arose during sample processing and should be investigated.

##### Turn-Around Time Check

One of the pitfalls of NGS is the turn-around time for HIVDR testing. While specimen receipt to generating results can happen in three days for Sanger sequencing, in our experience NGS-based HIVDR genotyping can take at least five to six days to complete. It is critical to check if any samples are taking too long to be reported. Many reasons can be attributed to this, such as if the specimen have missed a scheduled batch test, if it experienced a failed test, if it was mixed up with another sample, or if there was an issue with an instrument. Without systematically tracking turnaround time, labs have no way of knowing whether there are issues.

#### 2.2.4. Data Review, Results Authorization, and Release

While an LIS is important for keeping a repository of analytical results, it should also serve as a one-stop shop for data visualization, review and authorization from the various tools in the system. Details on bioinformatics parameters or configuration settings, reference datasets, batch information, controls on the experiment, sequencing, bioinformatics QC, instrument specifications, and reagent information should be accessible at different levels of result certification. The system should also be able to use autovalidation of results, which limits human intervention and increases efficiency in laboratory operations [51,52].

### 2.3. Post-Analytical

To enhance an institutions ability to report diagnostic results to clients or external surveillance systems in a timely fashion adoption of data standards would be desirable, but is not currently widespread. The integration with other ehealth systems requires the adoption of common electronic terminology such as Logical Observation Identifiers Names and Codes (LOINC), Systematized Nomenclature of Medicine Clinical Terms (SNOMED CT), and pCLOD (Canadian version of LOINC) ensuring systems can both transmit and receive messages. Without human intervention, results can be coded, transmitted, parsed and incorporated between client and testing laboratories.

While an LIS collects and stores data, an effective business intelligence tool (BI), such as Power BI (Microsoft, Redmond, WA, USA) or Cognos (IBM, Armonk, NY, USA) allows lab managers and those responsible for testing to further investigate the data. The evolution of artificial intelligence will only improve this as we begin to learn to model and code systems to look for trends in the data to flag potential quality control issues and/or health events of interest. It also allows the lab to perform various quality checks on performance metric trends, including the following functionalities:

1. QC results displayed in Levey–Jennings plots and flag for violations according to Nelson or Westgard rules [11,53];
2. Performance comparison of different reagent lot numbers, equipment, operators, and test controls run in different batches. A useful control to monitor is a mixture of clonal samples with known nucleotide mixtures and the comparison of the frequency of those mixtures. A display of histograms of mutations in test controls or repeated samples with definable flags of signification deviation from historic mutation frequencies;
3. Automated scheduling of equipment maintenance and alerts staff of appropriate QC tasks;
4. Automated tracking and stock management of reagent and consumables;
5. Automated notification to lab manager of specimens with increased turnaround time;
6. Automated notification of low specimen volumes and identified bottle necks;
7. The system should allow monitoring of equipment performance, such temperature logs, frequency of failed runs, environmental conditions, and any documentations required by accrediting bodies;
8. The system should provide summaries of QC reports to supervisors for review, corrective and preventive actions;
9. Trend interesting results that are of interest to public health, such as the identification of genetic or transmission clusters, or changes in the prevalence of certain drug-resistant mutations [54].

An effective LIS also requires dedication from laboratory management and dedicated local IT personal to develop and maintain not only the LIS and the various databases but also the infrastructure that houses them. By having in-house support from IT, the laboratory can:

1. Frequently update or investigate new bioinformatics software which cannot be locked down to traditional Information Technology (IT) change control processes often associated with universal software applications used in office settings;
2. Have IT security experts imbedded within scientific computers to ensure the hardware and software are secure, protected and monitored against threats that could compromise the security of the data they hold;
3. Facilitate evolution of laboratory test for HIV drug resistance. Changes in the status quo often require a business analyst, programmer and infrastructure personnel to analyze the requirement, develop/modify the application and maintain the infrastructure without impacting business continuity;
4. Reduce licensing costs by eliminating redundant LIS in an organization.

## 3. Discussion

Next-generation sequencing platforms have been increasingly implemented in genomics laboratories for the purposes of higher throughput data generation, potential reduction in costs driven by massive batch testing, and higher resolution of data analysis to detect minor variants. Generally, guidelines for NGS-based diagnostics in clinical laboratories are being developed based on regulation implemented by the Clinical Laboratory Improvement Amendments (CLIA) in the United States [55]. In April 2018, the Food and Drug Administration released a guidance document on considerations for NGS-based diagnosis of germline diseases in efforts of accelerating regulatory practices for NGS [56]. However, because of the complexities of NGS technologies and their wide range of applications to different diseases, there lacks a consensus regarding performance thresholds to detect minor variants and reference materials to evaluate these assessments [37]. With HIV, clinically relevant mutation thresholds are still being debated, as lower thresholds have higher sensitivity in detecting virological failure but with a cost of the inability to identify people with viral suppression [57]. Furthermore, because of the uncertainty of detecting low abundant mutations, it is recommended that Sanger-like thresholds are implemented for routine clinical and surveillance testing of HIV-1 drug resistance (WHO/HIVResNet HIV Drug Resistance Laboratory Operational Framework December 2019, in preparation). Here, we recommend quality control measures to guide NGS-based HIV drug resistance genotyping in a clinical setting using 20% mutation threshold in the framework of a laboratory information management system. The performance metrics discussed in this paper are used as guidelines as various assays may adhere to different quality control parameters.

Incorporation of a LIS in a clinical lab is crucial for following the life cycle of a specimen, especially in the context of next-generation sequencing, whereby the data produced is large and complex. While incorporation of a LIS may be too costly for clinical labs in low- and middle-income countries, an open-source web-based laboratory information management system (MendeLIMS) has been developed for continuously evolving protocols of next-generation sequencing technologies [58]. User-friendly web-based HIVDR bioinformatics pipelines, as HyDRA [24], PASeq [26], and EXATYPE [27], can be used along with MendeLIMS to negate the need for in-house bioinformaticians. Recently, quality assurance on NGS data in clinical and public health settings have been thoroughly discussed [6,8,59,60,61,62], however these studies are not specific to HIV drug resistance testing and they do not address quality control measures applied on the final sequencing data (i.e., consensus sequence and variant calls).

Because of how NGS is performed, such as library preparation in a 96-well open-plate format, many steps of amplification, pooling multiple samples for multiplexing in one tube, and its intrinsically high sensitivity in detecting minor variants, it is increasingly important to control for contamination and sequence artefacts. Detecting significant sample carry-over is relatively quick and simply performed by enumerating the frequency of mixed nucleotide bases in a consensus sequence. The identification of minor carry-overs that may appear as low frequency mutations can be computationally taxing with a significant increase in run time that may delay drug resistance reporting. The current guideline of detecting cross-contamination for Sanger-based HIVDR testing is to include sequences derived from other batches (within 3 months of testing) to be included in the quality control step, however this may further increase computing time for NGS reads [30]. Certain strategies have been previously implemented, such as the removal of reads that have better mapping metrics to other samples’ consensus sequence [38,40], filtering reads based on Hamming distance distribution [39], or blacklisting infrequent “subgraphs” or “co-localized” variants in a phylogenetic tree from further analyses [63,64]. With the exception of phyloscanner, a problem may arise with these methods if a host is infected with multiple viral strains. None of the current HIVDR pipelines have implemented cross-contamination checks to ensure data generation of about 1 h. Studies have yet to compare these different ways of detecting low level contaminants and their respective processing times. The frequency of these small sample carry-overs in a given assay and their significance in contributing to variant calling and drug resistance mutations also has yet to be determined.

## 4. Conclusions

Next-generation sequencing technologies are being utilized for HIV drug resistance testing for high resolution genotyping, resolving ambiguous base calls, and potential cost savings. However, NGS challenges the status quo in a clinical setting where a flexible laboratory information system is required, as NGS-based protocols evolve quickly. While previous studies discussed the implementation strategies of NGS into clinical and public health settings, here we attempt to shed light on the role of incorporating an LIS for Quality Control on the final sequencing result, and monitor each of the steps that went into creating it.

## Figures and Tables

**Table 1:** A summary of performance metrics and thresholds at each quality control checkpoints.

Metric/Threshold	Sample Expected Value	Sample QC Tool
*QC1: Post-PCR*		
Amplicon	Negative control: no bandPositive control: band at correct size	Gel/Capillary electrophoresis
*QC2: Library Preparation*		
Library size	Normal distribution around 300–500 bp	Bioanalyzer/Tapestation ^1^
Library concentration	0.2 ng/μL	Bioanalyzer/Tapestation
*QC3: Post-Sequencing Run*	See Hutchins et al. [8]	SAV ^2^
*QC4: Pre-processing*	See Hutchins et al. [8]	FastQC ^3^
*QC5: Post-Reference Mapping* *(performed after final remapping)*		
Sequence Coverage	PR: codon 10–93 RT: codon 41–238 IN: codon 51–263	HIVDR Pipeline, Tablet ^4^, UGENE ^5^
Mean read depth	≥1000	HIVDR Pipeline, Tablet, UGENE
*QC6: Mislabeling/Contamination* *(Check for genetic relatedness)*		
Nucleotide mixture	<3.5% nucleotide positions	MEGA ^6^
Sequences from same patient	<2.5% genetic dissimilarity	WHO BCCFE HIVDR QC ^7^
Intra-batch sample vs other sample	≥0.5% genetic dissimilarity	WHO BCCFE HIVDR QC
Sample vs control strain	≥0.5% genetic dissimilarity	WHO BCCFE HIVDR QC
Across-batch sample vs other sample	≥0.5% genetic dissimilarity	WHO BCCFE HIVDR QC
*QC7: “Bad” Mutations/Variant Calls*		
“Unusual” mutations	<1.0%	HIVdb-NGS ^8^
Signature APOBEC hypermutations	<3	HIVdb-NGS
APOBEC-context DRMs	<2	HIVdb-NGS
Stop codons	0	HIVdb-NGS
Codon insertion/deletion	0	HIVdb-NGS
Frameshift insertion/deletion	0	HIVdb-NGS
*Variant Calling*		
Position depth	≥100 reads	HIVDR Pipeline
Q score	Q≥30	HIVDR Pipeline
Variant count	≥5 reads	HIVDR Pipeline
Turnaround Time	5–6 Days	N/A

^1^ Bioanalyzer or Tapestation (Agilent Technologies, Santa Clara, CA, USA); ^2^ Sequence Analysis Viewer (Illumina, San Diego, CA, USA);^; 3^ FastQC (Babraham Institute, Cambridge, UK); ^4^ Tablet (The James Hutton Institute, Aberdeen, UK); ^5^ UGENE (Unipro, Novosibirsk, Russia); ^6^ Molecular Evolutionary Genetics Analysis (Temple University, Philadelphia, PA, USA); ^7^ WHO BCCFE HIVDR QC Tool (University of British Columbia, Vancouver, BC, Canada); ^8^ HIVdb-NGS (Stanford University, Palo Alto, CA, USA).
